# Severe bone marrow edema on sacroiliac joint MRI increases the risk of low BMD in patients with axial spondyloarthritis

**DOI:** 10.1038/srep22158

**Published:** 2016-03-02

**Authors:** Ha Neul Kim, Joon-Yong Jung, Yeon Sik Hong, Sung-Hwan Park, Kwi Young Kang

**Affiliations:** 1 Division of Rheumatology, Department of Internal Medicine, College of Medicine, The Catholic University of Korea, Seoul, South Korea; 2 Department of Radiology, College of Medicine, The Catholic University of Korea, Seoul, South Korea; 3 Division of Rheumatology, Department of Internal Medicine, Incheon St. Mary’s Hospital, The Catholic University of Korea, Incheon, South Korea

## Abstract

To determine the association between inflammatory and structural lesions on sacroiliac joint (SIJ) MRI and BMD and to identify risk factors for low BMD in patients with axial spondyloarthritis (axSpA). Seventy-six patients who fulfilled the ASAS axSpA criteria were enrolled. All underwent SIJ MRI and BMD measurement at the lumbar spine, femoral neck, and total hip. Inflammatory and structural lesions on SIJ MRI were scored. Laboratory tests and assessment of radiographic and disease activity were performed at the time of MRI. The association between SIJ MRI findings and BMD was evaluated. Among the 76 patients, 14 (18%) had low BMD. Patients with low BMD showed significantly higher bone marrow edema (BME) and deep BME scores on MRI than those with normal BMD (p < 0.047 and 0.007, respectively). Inflammatory lesions on SIJ MRI correlated with BMD at the femoral neck and total hip. Multivariate analysis identified the presence of deep BME on SIJ MRI, increased CRP, and sacroiliitis on X-ray as risk factors for low BMD (OR = 5.6, 14.6, and 2.5, respectively). The presence of deep BME on SIJ MRI, increased CRP levels, and severity of sacroiliitis on X-ray were independent risk factors for low BMD.

Axial spondyloarthritis (axSpA) is a chronic inflammatory disease mainly affecting the sacroiliac joints (SIJ) and the vertebrae. The disease includes ankylosing spondylitis (AS) with definite radiographic sacroiliitis and non-radiographic axSpA (nr-axSpA), which shows no definitive evidence of radiographic sacroiliitis. Patients with axSpA have an increased risk of low bone mass. AS patients have a high prevalence of osteoporosis^1^, which is observed in both early and advanced cases^2^,^3^,^4^,^5^. A recent study showed that non-radiographic (nr)-axSpA patients show significantly greater bone loss than patients with mechanical back pain^6^. This increased risk is related to high disease activity, pro-inflammatory cytokines, and defects in mechanical and mineralization due to subclinical gut inflammation^1^,^7^,^8^. Systemic inflammation may play a critical role in the pathogenesis of osteoporosis in axSpA. Acute-phase reactants, which indicate systemic inflammation, show a good correlation with bone mass^3^,^9^,^10^. Under inflammatory conditions, tumor necrosis factor alpha (TNF-α) and IL-6 amplify osteoclastogenesis by inducing the expression of receptor activator of nuclear factor-KB ligand (RANK-L), leading to generalized osteoporosis^11^.

Bone inflammation in axSpA patients may be the major cause of systemic inflammation, leading to increased bone resorption and low BMD. Bone marrow edema (BME) on MRI reflects acute inflammatory changes in the bone. BME is detected as areas of increased signal intensity in T2-weighted images with fat saturation or short I inversion recovery sequences, and is interpreted as an acute inflammatory lesion in axSpA^12^. A recent study revealed the association between BME on lumbar spinal MRI and BMD in nr-axSpA^6^. Another study in patients with early inflammatory back pain showed that the presence of BME on lumbar spine MRI was associated with low BMD^13^. These findings suggest that bone inflammation is associated with bone loss.

BME on SIJ MRI reflects site-specific inflammation in the SIJ. A longitudinal study in patients with undifferentiated SpA showed that BME on SIJ MRI was associated with femoral neck BMD after 12 months^14^. On the other hand, in patients with early inflammatory back pain, BME on SIJ MRI is not associated with low BMD after adjusting for confounding factors^13^. Two previous studies of the association between SIJ MRI findings and BMD in patients with inflammatory back pain evaluated only the presence/absence of BME^13^,^14^. Furthermore, no study has examined the association between the severity of bone inflammatory or structural lesions on SIJ MRI and BMD in axSpA patients fulfilling the ASAS criteria. Therefore, the aims of the present study were to determine the association between acute inflammatory lesions and structural lesions on SIJ MRI and BMD, and to evaluate the risk factors for low BMD in axSpA.

## Results

### Characteristics of the study population

Data from 76 patients (80% male) were analyzed. The mean age was 33 ± 12 years (range, 19–50) and the mean symptom duration and time after diagnosis of axSpA were 5.5 ± 8.1 and 1.5 ± 3.1 years, respectively. Twenty-four were current smokers (32%) and 87% were HLA-B27-positive. Patients showed high disease activity, with a mean ASDAS-ESR of 2.9 ± 1.0 and ASDAS-CRP of 2.6 ± 1.1, and a mean BASDAI of 4.5 ± 2.0. The mean mSASSS was 5.2 ± 10.9 and 58% had definite radiographic sacroiliitis.

Among the 76 patients, 14 (18%) showed low BMD (Z ≤ −2 at any site). Table 1 compares the characteristics of the patients with and without low BMD. There were no differences between patients with normal BMD and those with low BMD in terms of demographic data, disease duration, HLA-B27 positivity, or social habits. There were no differences in the levels of BALP and sCTX between the two groups. The grade of sacroiliitis on X-ray and mSASSS was lower in patients with normal BMD (p = 0.015 and 0.022, respectively).

Table 2 compares the acute inflammatory scores and structural scores on SIJ MRI for axSpA patients with or without low BMD. The mean BME score on six slices was higher in patients with low BMD (p = 0.047). The mean depth score (BME ≥ 1 cm from the SI joint surface) was also higher in patients with low BMD (p = 0.007). However, there were no differences in the structural lesion scores between the two groups.

Table 3 lists the r coefficients for the correlation between acute inflammatory findings on SIJ MRI, laboratory data, and BMD. The scores for BMD and deep edema correlated with sCTX. The BME score showed a significant correlation with the femoral neck Z score, and total hip BMD, T score, and Z score. The depth score also correlated with the femoral neck T and Z scores, and with total hip BMD and Z score. The ESR and CRP levels did not correlate with bone turnover markers, but the ESR did correlate with BMD, T score, and Z score at all sites. CRP also showed a significant correlation with BMD and the T score and Z score at each site, but not with the femoral neck Z score. In contrast to acute inflammatory lesions, structural lesions on SIJ MRI did not correlate with variables associated with bone density (Supplemental Table 1).

Subgroup analysis of patients with symptom duration of 1 year or less and those with symptom duration greater than 1 year was also performed. Table 4 shows the correlation between the scores for inflammatory lesions on SIJ MRI and variables associated with systemic inflammatory markers and total hip BMD in the two groups, stratified according to symptom duration. The depth scores for BME on MRI in patients with short symptom duration correlated with hip BMD, the T score, and the Z score; however, this was not the case for patients with symptom duration greater than 1 year. ESR was negatively correlated with total hip variables in both groups.

**Figure 1:**
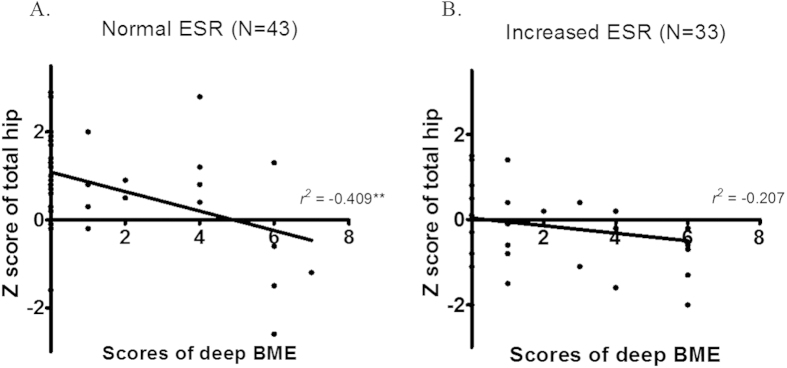
Correlation between depth scores for bone marrow edema on sacroiliac MRI and the total hip BMD Z score. The bone marrow edema depth scores on SIJ MRI correlated with the total hip Z score for the normal ESR group (n = 43) (**A**), but not with that for the elevated ESR group (n = 33) (**B**).

The Fig. 1 shows the relationship between the Z score for the total hip and the BME depth scores in patients with a normal or elevated ESR. When patients were stratified according to the ESR value (cut-off, 20 mm/hr), the BME depth scores on SIJ MRI correlated with the total hip Z score in the normal ESR group but not in the elevated ESR group.

Table 5 shows the results of univariate and multivariate analyses of low BMD (Z score ≤ −2, at any site). Univariate logistic regression analysis identified the presence of deep BME on SIJ MRI, elevated ESR and CRP, grade of radiographic sacroiliitis, and spinal radiographic progression (mSASSS ≥ 2) as being associated with low BMD. Multivariate logistic regression with forward selection of variables statistically associated with low BMD in univariate analysis identified the presence of deep BME on SIJ MRI as a risk factor for low BMD (OR: 5.6; 95% CI: 1.1–27.9, P = 0.036). Increased CRP (OR: 14.6; 95% CI: 2.5–85.0; p = 0.003) and grade of radiographic sacroiliitis on X-ray (OR: 2.5; 95% CI: 1.1–5.8; p = 0.036) were also associated with low BMD.

## Discussion

This cross-sectional study examined the association between acute inflammatory and structural lesions on SIJ MRI and BMD in patients with axSpA. Active bone inflammation at the SIJ was positively correlated with the expression of bone resorption markers and was associated with BMD in axSpA patients. The results also showed that severe acute sacroiliitis on MRI, increased CRP levels, and radiographic sacroiliitis are associated with an increased risk of low BMD.

Here, we found that 18% of patients had low BMD, despite their young age (mean, 33 ± 12 years). Disease activity in axSpA contributes to the rate of bone loss, and osteoporosis is considered a manifestation of the disease itself rather than a comorbidity^15^. The increased risk of low BMD in patients with inflammatory back pain cannot be explained by traditional risk factors for osteoporosis^13^. Also, we observed no differences in known risk factors for low BMD, such as age, body mass index, smoking, and alcohol excess, between patients with or without low BMD. This may be because increased bone resorption due to inflammation is the main cause of secondary osteoporosis in patients with chronic inflammatory arthritis^2^. Previous studies report that systemic inflammation (as assessed by the ESR or CPR levels) is related to low BMD in axSpA^1^,^3^,^9^.

Recent studies show that bone inflammation, as assessed by MRI, is associated with low BMD in patients with nr-axSpA and IBP^6^,^13^,^14^. These studies report that the presence of BME on MRI is the main risk factor associated with low BMD. To date, only two studies have reported an association between the presence of BME on SIJ MRI and BMD^13^,^14^ in patients with early inflammatory back pain. These studies included patients with IBP that did not fulfil the ASAS criteria. No study has yet examined only patients that fulfil the ASAS criteria.

This is the first study to report an association between quantified findings on SIJ MRI and BME in axSpA patients. The results confirm that axSpA patients showing bone inflammation at the SIJ are at increased risk of low BMD. Among the inflammatory SIJ lesions that can be detected on MRI, only BME is considered reliable for identifying active sacroiliitis^16^. BME is thought to reflect the presence of osteitis and is associated with histological inflammation and radiographic progression^17^. Sacroiliac biopsy specimens from SpA patients with MRI-detected sacroiliitis show an inflammatory infiltrate comprising T cells, macrophages, and scarce B cells^18^. Here, we quantified active sacroiliitis using the SPARCC method^19^, which involves scoring BME on six coronal slices at the SIJ. BME is scored according to its presence, intensity, and depth. The depth of BME is defined as positive when 1 cm or more of continuous edema extends in a horizontal direction away from the articular surface. Each SIJ is evaluated as a whole. The depth associated with any part of a joint receives a score of 1. The depth of BME across six slices ranged from 0 to 12^19^. BME indicates the presence of acute inflammation, and increased density and depth indicate severe acute inflammation. We found that the severity of BME was negatively correlated with bone loss at the hip and femoral neck.

We found it interesting that severe BME was negatively correlated with the hip Z score in patients with normal ESR levels. Multivariate analysis also showed that severe BME was independently associated with low BMD. Thus, both bone inflammation and systemic inflammation play a role in the increased risk for low BMD in axSpA. These data suggest that BMD should be measured in axSpA patients with normal systemic inflammatory levels (as well as in those with an active systemic inflammatory condition) if they have severe active sacroiliitis on SIJ MRI.

Multivariate analysis showed that the severity, rather than the presence, of BME was associated with low BMD. The relationship between the BME at the SIJ and BMD was stronger in patients with early axSpA (symptom duration ≤1 year) than in patients with longer symptom duration. One possible explanation for this is that sacroiliitis is a main source of systemic inflammation during the early phase of the disease, whereas the influence of other inflammatory sources, such as the spine or peripheral joints, may increase during the advanced phase.

A large cohort study examining the association between BME on MRI and BMD in subjects with IBP (71% of whom met the ASAS criteria for axSpA) showed that low BMD was associated with the presence of BMD (spine or SIJ), increased ESR or CRP, and male gender^13^. However, although univariate analysis identified BME on SIJ MRI as being associated with low BMD, multivariate analysis did not. Another study of patients with IBP (83% of whom met the ASAS criteria for axSpA) showed that the presence of BME on baseline SIJ MRI was associated with bone loss at the femoral neck only after 12 months, whereas baseline CRP levels were associated with bone loss at both the spine and hip^14^. The results of the present study also showed that inflammatory findings on SIJ MRI correlated with BMD at the femoral neck and total hip, but not with that in the lumbar spine.

The present study also showed that serum CTX-I levels correlated with the severity of active sacroiliitis on MRI. This suggests that active sacroiliitis results in bone loss through an increase in bone resorption. However, we did not find that sCTX-I correlated with markers of systemic inflammation. Some studies show that sCTX correlates with CRP levels^20^,^21^; therefore, the relationship between inflammatory markers and bone resorption markers should be confirmed in further large scale longitudinal studies.

We also found that the grade of radiographic sacroiliitis on X-ray was a risk factor for low BMD, but radiographic spinal progression was not a significant factor in multivariate analysis. The total score for acute inflammatory lesions and the BME score correlated with both the ESR and CRP levels (data not shown). The severity of sacroiliitis on X-ray may reflect cumulative inflammation at the SIJ, suggesting that cumulative bone inflammation at the SIJ, as well as acute sacroiliitis, may be associated with low BMD.

This study has several limitations. The study was of cross-sectional design; therefore, the results should be interpreted with a degree of caution. The status of BME on MRI may only reflect inflammatory status at the time of MRI measurement. Longitudinal studies may be a more precise method of assessing whether sacroiliitis on SIJ MRI is associated with BMD. Another limitation is that we did not measure spinal inflammation. We found no association between sacroiliitis on MRI and lumbar BMD. Therefore, bone loss at the spine of axSpA patients may be due to the localized effects of inflammation.

In conclusion, the present study is the first to examine the association between quantified active sacroiliitis on MRI and BMD in axSpA patients that fulfilled the ASAS criteria. Acute inflammatory lesions, but not structural lesions, on SIJ MRI were associated with low BMD. The presence of deep BME on SIJ MRI, increased CRP levels, and the severity of sacroiliitis on X-ray were independent risk factors for low BMD in patients with axSpA.

## Patients and Methods

### Patients

Between August 2013 and May 2015, 76 consecutive axSpA patients from Incheon Saint Mary’s Hospital were recruited to this cross-sectional study. All enrolled patients fulfilled the imaging arm of the Assessment of SpondyloArthritis international Society (ASAS) axSpA criteria^12^. Enrolled patients underwent baseline MRI scans of both SIJs. BMD was measured using Dual-energy X-ray absorptiometry (DXA) at the time of MRI. All patients were aged between 20 and 50 years. Patients with thyroid or parathyroid disorders and those with chronic renal or liver disease were excluded. All subjects provided informed consent. The study was approved by the ethics committee at Incheon Saint Mary’s Hospital, Catholic University of Korea (XC13RIMI0129O) and conducted in accordance with the ethical guidelines set down in the Declaration of Helsinki (1975).

### Clinical, laboratory, and radiologic assessment

Demographic data included age, sex, time after symptom onset, years from diagnosis of AS, the presence of HLA-B27, smoking status, family history, the presence of peripheral arthritis, and the use of medications such as anti-inflammatory drugs (NSAIDs) and sulfasalazine. Risk factors associated with low BMD included smoking status, excess alcohol (≥3 units daily), height, weight, body mass index (BMI) (kg/m^2^), and menopause. Measures of disease activity were collected using the Bath Ankylosing Spondylitis Disease Activity Index (BASDAI)^22^. All scores were recorded on a visual analogue scale from 0 to 10. The Bath Ankylosing Spondylitis Functional Index (BASFI)^23^ score and the number of swollen and tender joints were also recorded. The Ankylosing Spondylitis Disease Activity Score (ASDAS) was calculated using different formulae, as described previously^24^.

Laboratory assessments were performed at the time of the MRI assessment. The presence of the HLA-B27 was also assessed. The erythrocyte sedimentation rate (ESR) (mm/hr) and CRP (mg/l) levels were measured and bone turnover was studied by measuring the levels of bone resorption markers and serum levels of cross-linked telopeptide of type-I collagen (sCTX) and bone-specific alkaline phosphatase (BALP). sCTX was measured in an electrochemiluminescence immunoassay (ECLIA; Elecsys 2010 Roche Diagnostics, Mannheim, Germany), according to the manufacturer’s protocol. Serum BALP was measured in an enzyme immunoassay (Micro Vue BAP; Quidel, San Diego, USA), according to the manufacturer’s protocol.

For all patients, radiographs of the cervical spine, lumbar spine, and pelvis were obtained at the time of the MRI assessment. Lateral views of the cervical and lumbar spine were scored according to the modified Stoke AS Spinal Score (mSASSS)^25^. Sacroiliitis was scored from right- and left-sided pelvic radiographs using the modified New York criteria^26^. The average score for both sides was used for analysis. Sacroiliitis and the mSASSS were scored by a single trained expert (HN Kim), who was blinded to the patient characteristics.

### MRI protocol

MRI of the SIJ was performed at baseline. Images were obtained using a 3.0 T MRI unit (Verio/Skyra; Siemens Medical, Erlangen, Germany) and a body flexed array coil (Siemens Medical, Erlangen, Germany). Assessment of structural lesions in the SIJ was based on T1-weighted turbo spin echo (TSE) MRI sequences. Assessment of inflammatory lesions was based on T2-weighted FS TSE sequences. The sequence protocols were as follows: semi-coronal (along the long axis of the sacral bone) TSE (slice thickness (ST) 3 mm; repetition time/echo time (TR/TE) 636/11 ms), and semi-coronal T2-weighted FS TSE (ST 3 mm; TR/TE 5210/55 ms).

### Semi-quantitative assessment of MRI findings

Inflammatory and structural lesions on SIJ MRI were scored according to the SPondyloArthritis Research Consortium of Canada (SPARCC) method^19^,^27^. All scores were measured by an experienced musculoskeletal radiologist (JY Jung) who was blinded to the patient characteristics. Assessment of inflammatory lesions in the SIJs was based on T2-weighted FS TSE images. The SIJs were scored using the SPARCC method^19^ based on six consecutive coronal slices depicting the synovial portion of the joint. The scoring ranges were as follows: BME, 0–48; depth, 0–12; and intensity, 0–12. The maximum score is 72. Structural lesions on SIJ MRI were scored according to standardized definitions using the SPARCC SI structural lesion score (SSS)^27^, which is a semi-quantitative scoring method. The SSS was based on T1-weighted TSE images, with slices selected according to well-defined anatomical principles. Scoring was dichotomous (lesion present/absent) and based on five consecutive slices through the cartilaginous portion of the joint. The scoring ranges were as follows: fat metaplasia, 0–40; erosion, 0–40; backfill, 0–20; and ankylosis, 0–20. The maximum score is 120.

### BMD measurement

BMD (g/cm^2^) at the anterior-posterior lumbar spine (L1–L4) and femoral neck was measured using DXA (Lumbar Prodigy densitometer; Madison, WI, USA). All measurements were taken by an experienced operator using the same machine and standardized procedures with respect to participant positioning. BMD measured in the lumbar spine (L1–L4), left hip, and left femoral neck was expressed as follows: the number of grams of bone mineral per square centimeter (g/cm^2^), T score, and Z score. In the present study, all female patients were premenopausal and all male patients were under 50 years old. A position statement by the International Society for Clinical Densitometry (ISCD) recommends that Z scores be calculated in females prior to menopause and in males aged less than 50 years. A Z score ≤ −2.0 is defined as “below the expected range for age” and a Z-score > −2.0 is “within the expected range for age”. According to ISCD recommendations, a low BMD is defined as a Z score ≤ −2.0 SD (compared with the age-matched mean)^28^. Data were compared with the manufacturer’s reference values.

### Statistical analysis

Continuous data were expressed as the mean ± SD and categorical data as percentages. Continuous variables were compared using the Mann-Whitney U test and categorical variables were compared using the Chi-squared test. Spearman’s correlation coefficient was used to analyze correlations between variables. Multivariate logistic regression analysis was performed to ascertain the association between SIJ MRI findings and low BMD after adjusting for potential confounders. All variables with a p value < 0.10 in univariate analysis were incorporated as explanatory variables. Variables with a p value < 0.05 were entered into multivariate stepwise regression analyses (forward selection), whereas those with a p value > 0.1 were eliminated. A p value < 0.05 was considered statistically significant. Statistical analyses were performed using PASW statistics 18 (SPSS Inc., Chicago, IL, USA).

## Additional Information

**How to cite this article**: Kim, H. N. *et al*. Severe bone marrow edema on sacroiliac joint MRI increases the risk of low BMD in patients with axial spondyloarthritis. *Sci. Rep*. **6**, 22158; doi: 10.1038/srep22158 (2016).

## Supplementary Material

Supplementary Information

## Figures and Tables

**Table 1:** Characteristics of axial spondyloarthritis patients with normal and low bone mineral density (Z score ≤ −2 at any site).

Characteristic (N (%) or mean ± SD)	Patients with normal BMD (N = 62)	Patients with low BMD (N = 14)	*p* value
Age (years)	33.4 ± 12.1	29.0 ± 10.4	0.266
Male	47 (76)	14 (100)	0.059
Smoking, current	20 (32)	4 (29)	1.000
Alcohol excess	5 (8)	0 (0)	0.577
BMI, kg/m^2^	23.4 ± 3.8	23.4 ± 3.8	0.915
Time from symptom onset (years)	5.6 ± 8.4	5.3 ± 6.7	0.767
Duration since diagnosis (years)	1.7 ± 3.3	0.7 ± 1.9	0.244
Family history of axSpA	8 (13)	1 (7)	1.000
HLA-B27 positive	52 (84)	14 (100)	0.193
Peripheral arthritis	15 (24)	4 (29)	0.740
Patient global assessment	5.0 ± 2.2	5.6 ± 2.1	0.211
BASDAI, score (range, 0–10)	4.5 ± 2.1	4.7 ± 1.4	0.695
BASFI, score	2.0 ± 2.2	1.8 ± 1.2	0.637
ASDAS-ESR	2.7 ± 1.1	3.4 ± 0.7	0.026
ASDAS-CRP	2.5 ± 1.1	3.3 ± 0.8	0.008
ESR, mm/h	21.2 ± 20.7	37.5 ± 22.9	0.011
CRP, mg/l	9.2 ± 16.3	25.6 ± 26.4	0.002
Bone alkaline phosphatase (U/l)	25.6 ± 16.3	33.0 ± 14.1	0.092
sCTX (ng/ml)	0.4 ±0.4	0.4 ±0.3	0.485
Grade of sacroiliitis on X-ray	1.9 ± 1.0	2.7 ± 1.0	0.015
mSASSS	4.3 ± 14.2	8.9 ± 14.2	0.022
Number of syndesmophytes	1.5 ± 3.6	2.4 ± 5.1	0.653
Patients on NSAIDs	58 (95)	14 (100)	1.000
Patients on sulfasalazine	15 (25)	4 (29)	0.743
Lumbar spine BMD, g/cm^2^	1.20 ± 0.16	0.90 ± 0.09	<0.001
Lumbar spine T score	0.22 ± 1.32	−2.4 ± 0.80	<0.001
Lumbar spine Z score	−0.08 ± 1.15	−2.69 ± 0.44	<0.001
Femoral neck BMD, g/cm^2^	1.00 ± 0.15	0.87 ± 0.13	0.003
Femoral neck T score	0.31 ± 1.18	−0.94 ± 1.03	0.001
Femoral neck Z score	0.26 ± 1.09	−1.04 ± 0.83	<0.001
Total hip BMD, g/cm^2^	1.05 ± 0.15	0.90 ± 0.14	0.001
Total hip T score	0.75 ± 1.19	−0.72 ± 1.27	0.001
Total hip Z score	0.64 ± 1.12	−0.80 ± 1.03	<0.001

BMD, bone mineral density; BASDAI, Bath AS Disease Activity Index; BASFI, Bath Ankylosing Spondylitis Functional Index; ASDAS, Ankylosing Spondylitis Disease Activity Score; sCTX, serum cross-linked telopeptide of type-I collagen; mSASSS, modified Stokes Ankylosing Spondylitis Score; NSAIDs, nonsteroidal anti-inflammatory drugs.

**Table 2:** Sacroiliac joint MRI findings in patients with axial spondyloarthritis (N = 76).

MRI finding, (mean ± SD score)	Patients with normal BMD (N = 62)	Patients with low BMD (N = 14)	*p* value
Acute inflammation			
Bone marrow edema (0–48)	6.7 ± 8.1	10.1 ± 7.3	0.047
Depth (0–12)	1.3 ± 2.0	3.0 ± 2.6	0.007
Intensity (0–12)	0.8 ± 1.9	1.1 ± 1.8	0.196
Total score (0–72)	8.9 ± 10.9	14.4 ± 11.1	0.028
Structural lesions			
Fat metaplasia (0–40)	3.2 ± 5.7	5.9 ± 10.8	0.238
Erosion (0–40)	4.8 ± 4.3	7.2 ± 5.1	0.086
Backfill (0–20)	2.0 ± 2.5	3.8 ± 3.7	0.074
Ankylosis (0–20)	0.6 ± 2.1	2.1 ± 5.1	0.197
Total score (0–120)	1.5 ± 9.7	19.0 ± 14.2	0.008

**Table 3:** Correlation between acute inflammatory findings on sacroiliac joint MRI, inflammatory markers, bone turnover markers, and BMD in axSpA patients.

Variable(*r* coefficients)	Acute inflammatory lesion on SIJ MRI				Inflammatory marker	
	BME	Depth	Intensity	Total	ESR, mm/h	CRP, mg/l
sCTX (ng/ml)	0.266*	0.309**	0.131	0.283*	−0.156	−0.121
BALP (U/I)	−0.018	−0.002	−0.020	0.022	−0.067	0.022
Lumbar spine BMD, g/cm^2^	−0.139	−0.187	−0.020	−0.149	−0.485**	−0.419**
Lumbar spine T score	−0.145	−0.215	−0.006	−0.156	−0.466**	−0.424**
Lumbar spine Z score	−0.136	−0.212	−0.071	−0.146	−0.419**	−0.394**
Femoral neck BMD, g/cm^2^	−0.186	−0.197	−0.151	−0.202	−0.428**	−0.274*
Femoral neck T score	−0.249	−0.243*	−0.160	−0.235*	−0.393**	−0.260*
Femoral neck Z score	−0.218*	−0.298**	−0.183	−0.287*	−0.456**	−0.202
Total hip BMD, g/cm^2^	−0.237*	−0.233*	−0.174	−0.254*	−0.503**	−0.380**
Total hip T score	−0.268*	−0.205	−0.190	−0.233*	−0.456**	−0.360**
Total hip Z score	−0.341**	−0.330**	−0.233*	−0.345**	−0.419**	−0.309**

BMD, bone mineral density; SIJ, sacroiliac joint; BME, bone marrow edema; FM, fat metaplasia; sCTX, serum cross−linked telopeptide of thpe-1 collagen; BALP, bone-specific alkaline phosphatase.

*p < 0.05, **p < 0.01.

**Table 4:** Correlation between acute inflammatory findings on sacroiliac MRI, inflammatory markers, and total hip BMD (stratified according to symptom duration).

Variable(*r* coefficients)	Acute inflammatory lesion			Inflammatory marker	
	BME	Depth	Intensity	ESR, mm/h	CRP, mg/l
Sx. Duration ≤ 1 year (n = 35)					
Total hip BMD, g/cm^2^	−0.307	−0.438**	−0.052	−0.513**	−0.385*
Total hip T score	−0.253	−0.356*	−0.056	−0.407*	−0.382*
Total hip Z score	−0.311	−0.489**	−0.068	−0.419*	−0.311
Sx. Duration > 1 year (n = 41)					
Total hip BMD, g/cm^2^	−0.079	−0.003	−0.003	−0.517**	−0.347*
Total hip T score	−0.164	−0.099	−0.099	−0.501**	−0.311
Total hip Z score	−0.208	−0.136	−0.120	−0.443**	−0.270

BMD, bone mineral density; SJI, sacroiliac joint; BME, bone marrow edema.

*p < 0.05, **p < 0.01.

**Table 5:** Univariate and multivariate logistic regression analysis of low BMD (Z score ≤ −2 at any site).

Variable	Univariate analysis		Multivariate analysis	
	OR (95% CI)	*p* value	OR (95% CI)	*p* value
Presence of BME	6.2 (0.8–50.7)	0.089		
Presence of depth	5.4 (1.4–21.4)	0.016	5.6 (1.1–27.9)	0.036
Increased ESR (≥20 mm/hr)	6.7 (1.7–26.5)	0.007		
Increased CRP (≥5 mg/l)	13.6 (2.8–66.7)	0.001	14.6 (2.5–85.0)	0.003
Grade of sacroiliitis on X-ray	2.3 (1.2–4.4)	0.018	2.5 (1.1–5.8)	0.036
mSASSS (≥2)	3.8 (1.1–12.8)	0.032		

OR, odds ratio; CI, confidence interval; BME, bone marrow edema; mSASSS, modified Stokes Ankylosing Spondylitis Score.
