# Supplementary material for: Severe bone marrow edema on sacroiliac joint MRI increases the risk of low BMD in patients with axial spondyloarthritis
Source: Sci Rep. 2016 Mar 2;6:22158. doi: 10.1038/srep22158 (PMC4773924; doi:10.1038/srep22158)
Supplement: Supplementary Information [file srep22158-s1.doc]

**Severe bone marrow edema on sacroiliac joint MRI increases the risk of low BMD in patients with axial spondyloarthritis**

Ha Neul Kim^1†^, Joon-Yong Jung^2†^, Yeon Sik Hong^1, 3^, Sung-Hwan Park^1^, and Kwi Young Kang^1, 3*^

^1^Division of Rheumatology, Department of Internal Medicine, College of Medicine, The Catholic University of Korea, Seoul; ^2^Department of Radiology, College of Medicine, The Catholic University of Korea, Seoul; and ^3^Division of Rheumatology, Department of Internal Medicine, Incheon St. Mary's Hospital, The Catholic University of Korea, Incheon, South Korea

^†^These authors contributed equally to this work.

***Corresponding author:**

Kwi Young Kang, M.D., Ph.D.

Division of Rheumatology, Department of Internal Medicine, Incheon St. Mary's Hospital, College of Medicine, The Catholic University of Korea, #56, Dongsu-Ro, Bupyung-Gu, Incheon, South Korea.

Telephone: +82 32 280 5069; Fax: +82 32 280 5987; E-mail: kykang@catholic.ac.kr

**Supplement Table**. Correlation between structural findings on sacroiliac joint MRI and BMD in axSpA patients

| Variable  (*r* coefficients) |  | Structural lesion on SIJ MRI | | | | |
| --- | --- | --- | --- | --- | --- | --- |
|  |  | FM | Erosion | Backfill | Ankylosis | Total |
| Lumbar spine BMD, g/cm^2^ |  | 0.119 | -0.219 | -0.008 | 0.015 | -0.066 |
| Lumbar spine T score |  | 0.115 | -0.221 | -0.012 | 0.146 | -0.071 |
| Lumbar spine Z score |  | 0.007 | -0.174 | -0.022 | 0.105 | -0.008 |
| Femoral neck BMD, g/cm^2^ |  | 0.061 | -0.079 | -0.015 | 0.136 | -0.008 |
| Femoral neck T score |  | 0.053 | -0.090 | -0.058 | 0.140 | -0.032 |
| Femoral neck Z score |  | 0.026 | -0.116 | -0.018 | 0.138 | -0.037 |
| Total hip BMD, g/cm^2^ |  | 0.017 | -0.148 | -0.121 | 0.119 | -0.109 |
| Total hip T score |  | 0.027 | -0.135 | -0.144 | 0.157 | -0.089 |
| Total hip Z score |  | 0.009 | -0.190 | -0.149 | 0.123 | -0.130 |

BMD, bone mineral density; SJI, sacroiliac joint; BME, bone marrow edema; FM, fat metaplasia.

**p*<0.05, ***p*<0.01.
